# Complete Genome Sequence of *Faecalibacterium prausnitzii* Isolated from the Gut of a Healthy Indian Adult

**DOI:** 10.1128/genomeA.01286-17

**Published:** 2017-11-16

**Authors:** Satyabrata Bag, Tarini Shankar Ghosh, Bhabatosh Das

**Affiliations:** Molecular Genetics Laboratory, Centre for Human Microbial Ecology, Translational Health Science and Technology Institute, NCR Biotech Science Cluster, Faridabad, India

## Abstract

*Faecalibacterium prausnitzii* is the most abundant (~4%) member of the phylum *Firmicutes* found in the colon of healthy humans. It is a strict anaerobe and plays an important role in intestinal homeostasis. Here, we report the complete genome sequence of *F. prausnitzii* strain Indica.

## GENOME ANNOUNCEMENT

*Faecalibacterium prausnitzii*, a non-spore-forming and nonmotile bacterium closely related to members of *Clostridium* cluster IV, inhabits the colon of healthy humans and plays an important role in host physiology by modulating gut immunity and inflammation and by providing several metabolic functions to humans (1). Depletion of *F. prausnitzii* and reduced diversity of gut microbiota are often associated with the frequent incidence, prevalence, and severity of inflammatory bowel disease (2–4) and undernutrition (5). To understand the contribution of *F. prausnitzii* in host physiology, it is important to isolate commensal strains from different parts of the world and explore their genomic repertoire.

In the present study, we isolated *F. prausnitzii* strain Indica from the fecal sample of a healthy adult Indian subject. A fresh fecal sample was directly resuspended in prereduced phosphate-buffered saline, diluted, and plated on a trypticase soy agar plate (pH 7.0) supplemented with 5% (vol/vol) defibrinated sheep blood and 0.5 g/liter l-cysteine-HCl and 33 mM potassium acetate. Plates were incubated for 48 h at 37°C in an anaerobic workstation (Whitley DG250) filled with 80% N_2_, 10% CO_2_, and 10% H_2_. The genomic DNA of *F. prausnitzii* was extracted by a Translational Health Science and Technology Institute (THSTI) method after 48 h of incubation in rich medium under anaerobic growth conditions (6).

The complete genome sequencing of *F. prausnitzii* was done using Illumina (HiSeq 2500 System) and Oxford Nanopore Technologies (MinION) DNA sequencing platforms. Error-corrected long Nanopore and Illumina reads were used for the hybrid assembly with the SPAdes tool, which generated a single contig. The assembly was evaluated using Sanger sequencing. The assembled genome of *F. prausnitzii* strain Indica is 2,868,932 bp in length and has a GC content of 56.9%.

Analysis of the 2.86-Mb genome sequence of *F. prausnitzii* revealed 2,707 coding sequences, including 77 RNAs encoding genes. The genome is highly enriched with carbohydrate metabolic functions (215 genes). In addition to genes encoding central carbohydrate metabolism (63 genes), the *F. prausnitzii* genome contains a large number of genes encoding monosaccharide (50 genes), disaccharide and oligosaccharide (56 genes), and polysaccharide (9 genes) utilization functions. More than 14 genes in the *F. prausnitzii* genome are linked to fermentation functions that can efficiently ferment dietary carbohydrates into short-chain fatty acids, such as formate, acetate, propionate, butyrate, and mixed acids. Moreover, the genome of *F. prausnitzii* encodes several cofactors, vitamins, prosthetic groups, and functions for pigment biosynthesis (109 genes). Surprisingly, we also observed that the *F. prausnitzii* genome harbors 51 genes that can encode resistance against several antibiotics, including beta-lactams, fluoroquinolones, tetracyclines, aminoglycosides, and macrolides. Several efflux pumps (the major facilitator superfamily, the resistance-nodulation-cell division family, the small multidrug resistance family, the multidrug and toxic compound extrusion family, and the ATP-binding cassette family) encoding genes present in the genome of *F. prausnitzii* can contribute to antibiotic resistance and detoxification of xenobiotic compounds. The complete genome sequence of *F. prausnitzii* strain Indica will contribute to a better understanding of the biology of this commensal strain and the molecular basis of its dominance in the gut of Indian subjects.

### Accession number(s).

This whole-genome shotgun project has been deposited at DDBJ/ENA/GenBank under the accession number CP023819. The version described in this paper is the first version, CP023819.1.

## References

[1] Miquel S, Martín R, Rossi O, Bermúdez-Humarán LG, Chatel JM, Sokol H, Thomas M, Wells JM, Langella P 2013 *Faecalibacterium prausnitzii* and human intestinal health. Curr Opin Microbiol 16:255–261. doi:10.1016/j.mib.2013.06.003. 23831042

[2] Schirmer M, Smeekens SP, Vlamakis H, Jaeger M, Oosting M, Franzosa EA, Jansen T, Jacobs L, Bonder MJ, Kurilshikov A, Fu J, Joosten LA, Zhernakova A, Huttenhower C, Wijmenga C, Netea MG, Xavier RJ 2016 Linking the human gut microbiome to inflammatory cytokine production capacity. Cell 167:1125–1136.e8. doi:10.1016/j.cell.2016.10.020. 27814509 PMC5131922

[3] Maloy KJ, Powrie F 2011 Intestinal homeostasis and its breakdown in inflammatory bowel disease. Nature 474:298–306. 21677746 10.1038/nature10208

[4] Yatsunenko T, Rey FE, Manary MJ, Trehan I, Dominguez-Bello MG, Contreras M, Magris M, Hidalgo G, Baldassano RN, Anokhin AP, Heath AC, Warner B, Reeder J, Kuczynski J, Caporaso JG, Lozupone CA, Lauber C, Clemente JC, Knights D, Knight R, Gordon JI 2012 Human gut microbiome viewed across age and geography. Nature 486:222–227. 22699611 10.1038/nature11053 PMC3376388

[5] Ghosh TS, Gupta SS, Bhattacharya T, Yadav D, Barik A, Chowdhury A, Das B, Mande SS, Nair GB 2014 Gut microbiomes of Indian children of varying nutritional status. PLoS One 9:e95547. doi:10.1371/journal.pone.0095547. 24763225 PMC3999041

[6] Bag S, Saha B, Mehta O, Anbumani D, Kumar N, Dayal M, Pant A, Kumar P, Saxena S, Allin KH, Hansen T, Arumugam M, Vestergaard H, Pedersen O, Pereira V, Abraham P, Tripathi R, Wadhwa N, Bhatnagar S, Prakash VG, Radha V, Anjana RM, Mohan V, Takeda K, Kurakawa T, Nair GB, Das B 2016 An improved method for high quality metagenomics DNA extraction from human and environmental samples. Sci Rep 6:26775. doi:10.1038/srep26775. 27240745 PMC4886217

